# Near-unity charge readout signal in a nonlinear resonator without matching the sensor dissipation

**DOI:** 10.1038/s41467-026-75082-w

**Published:** 2026-07-02

**Authors:** Harald Havir, Andrea Cicovic, Pierre Glidic, Subhomoy Haldar, Sebastian Lehmann, Kimberly A. Dick, Ville F. Maisi

**Affiliations:** 1 NanoLund and Solid State Physics, Lund University, Lund, Sweden; 2 Institut Lumière Matière, UMR5306, CNRS, Université Lyon 1, Villeurbanne, France; 3 Department of Physics, Indian Institute of Technology Kanpur, Kanpur, Uttar Pradesh India; 4 Center for Analysis and Synthesis, Lund University, Lund, Sweden

**Keywords:** Quantum dots, Sensors, Superconducting devices, Techniques and instrumentation, Nonlinear phenomena

## Abstract

Dissipative sensors typically use linear resonators with impedance matching to achieve maximal signal and fast operation. The impedance matching, however, sets an upper limit to the bandwidth of the readout. In this paper, we present a nonlinear resonator performing the readout of a double quantum dot charge state via a charge-sensing quantum dot. We show that by driving the resonator in the nonlinear regime, we achieve a near-unity signal for a dissipative sensor. This despite not satisfying the sensor impedance matching requirements necessary for such large signals in the linear regime. Our experiments, supported by numerical calculations, demonstrate that the signal increase stems from the sensor dissipation shifting the onset of the nonlinear resonator response. By lifting the matching requirement, we open up an avenue to ultra-fast charge detectors as the resonator input-output coupling - setting the detector bandwidth - does not have to match to the typically much slower sensor dissipation rate.

## Abstract (web summary)

Fast readout in semiconductor quantum devices is limited by impedance-matching constraints. Here, the authors show that a nonlinear resonator converts sensor dissipation into a frequency shift, yielding near-unity charge-state contrast without matching and an order of magnitude faster readout of a double quantum dot.

## Introduction

Resolving the position of a single electron fast^1–3^ is a cornerstone in mesoscopic physics. It enables, for example, reading out spin qubits^4–8^, highly accurate metrology standards^9,10^, and probing electron transport statistics^11–14^. The fastest charge detection is currently achieved with dissipative sensors reaching near the quantum^1^ or shot-noise limit^15^. One of the key requirements for the fast dissipative sensors is that one uses a resonator circuit to match the sensor dissipation rate *κ*_s_ to the coupling rate *κ*_c_ between the readout line and the resonator circuit^3,16,17^.

Fulfilling this matching condition, *κ*_*c*_ = *κ*_*s*_, is equivalent of the usual impedance matching where the combined impedance of the sensor resistance and a lossless resonator circuit matches to the readout line impedance. This yields a unity change ∣Δ*r*∣ = 1, to the measured reflection coefficient *r* between the cases with and without the sensor dissipation *κ*_*s*_. If the resonator has any other spurious losses with a rate *κ*_*i*_, the response ∣Δ*r*∣ gets suppressed. Therefore the internal losses need to be low, *κ*_c_ ≫ *κ*_i_, to maintain the near unity change.

A detector operating under these conditions *κ*_s_ = *κ*_c_ ≫ *κ*_i_ thus achieves the unity readout signal enabled by impedance matching. Increasing the detection speed determined by the resonator linewidth requires therefore that both *κ*_s_ and *κ*_c_ are increased. Otherwise the readout signal is suppressed, which slows down the detection speed as longer averaging time is needed to reach sufficient signal-to-noise ratio.

In this paper, we show that the matching requirement can be circumvented. We use a nonlinear resonator to turn the dissipative response to a frequency shift, allowing us to use this shift similarly as done for inductive superconducting devices^18,19^, dispersive QD sensors^3,5,7,17^, and superconducting qubits^20,21^ that all have a frequency shifting response inherently. This results in a near-unity reflection response for the dissipative response despite not following the above matching requirements. We demonstrate further experimentally that the charge readout speed is increased by one order of magnitude in the nonlinear case, as compared to the corresponding linear case, and analyze that *κ*_s_ is not directly limiting the maximum attainable speed as is the case for the linear detection. Furthermore, the nonlinear operation mode allows for a protection scheme where two charge states are protected from the readout signal: for one of the states, the sensor is not conducting, achieving a protection the same way as spin qubit readout in refs. ^22,23^, and for the other, the nonlinearity shifts the resonator mode away from the frequency of the readout signal, hindering the readout signal to drive the resonator efficiently. The nonlinearities therefore enable both a higher readout bandwidth as well as new device concepts (the protection scheme and a way to circumvent the matching requirement and yet obtain a near-unity signal) for the dissipative sensors.

## Results

**Fig. 1:**
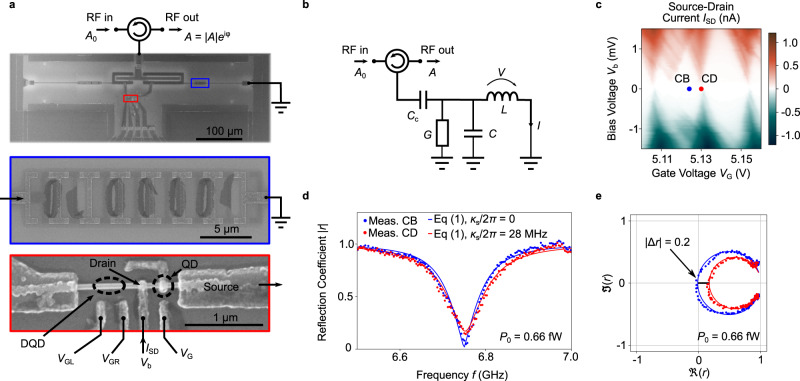
Device and its response in the linear regime. **a** Scanning electron micrographs of the measured device. The input/output port for RF signals at the top of the figure couples to the resonator via the longer c-shaped finger capacitor on the right. The inductance for the resonator is achieved with an array of *N* = 13 SQUIDs (blue) in series. An InAs nanowire with crystal-phase defined barriers contains the sensor QD and a DQD, with metallic connections defining the source- drain- and gate contacts to the QD and DQD. **b** A circuit model of the device. The input/output port couples to the resonator via a capacitance *C*_c_, which defines the resonance frequency together with the capacitance *C* to ground and inductance *L* of the SQUID-array. The QD adds a conductance *G*. **c** Measured current *I*_SD_ through the quantum dot as a function of the gate voltage *V*_G_ and bias voltage *V*_b_. An additional Coulomb diamond with excited level structure is also visible in the response. This is transport from the DQD which connects to the same dc measurement line. **d** Reflection coefficient ∣*r*∣ as a function of the drive frequency *f* in the linear regime at Coulomb blockade (CB, blue) and at Coulomb degeneracy (CD, red) with input power *P*_0_ = 0.66 fW. The solid lines are fits to Eq. (1). **e** The data of **d** plotted in the IQ-plane. The maximum difference in measured amplitude between the CB and CD cases is 0.2*A*_0_.

The measured device, presented in Fig. 1a, contains a QD-based charge sensor (red) in the same geometry as ref. ^24^ and a nonlinear resonator. The nonlinearities in the resonator are achieved with an array of *N* = 13 superconducting quantum interference devices (SQUIDs) in series (blue)^25–30^. As shown in the circuit diagram of Fig. 1b, this array yields an inductance *L* of the resonator that connects on one end to ground, while the other end is floating. The floating side therefore has an oscillating voltage *V* of the resonance mode. An in-going radio frequency (RF) readout signal with amplitude *A*_0_ couples to this voltage via a finger capacitor *C*_c_. The source contact of the sensor QD also connects directly to the voltage. As the drain side of the QD is shunted capacitively to ground, the QD transport conductance *G* adds dissipation directly into the resonator^3,31,32^.

Figure 1c presents the electrical transport of the sensor QD, measured in a dilution refrigerator with electronic base temperature *T* = 50 mK. Here we measure the electrical current *I*_SD_ as a function of DC bias voltage *V*_b_ and gate voltage *V*_G_. We see that at a gate voltage *V*_G_ = 5.13 V, the QD is conducting current with conductance *G* = 0.4 μS at the Coulomb degeneracy (CD) while at larger and smaller *V*_G_ the conduction stops due to the Coulomb blockade (CB). Figure 1d, e presents the corresponding resonator response, showing the measured resonator reflection coefficient ∣*r*∣ = ∣*A*/*A*_0_∣ as a function of the input drive frequency *f* in the linear regime at input power *P*_0_ = 0.66 fW. We see that making the QD conducting increases the linewidth of the response, along with a change in the reflection coefficient $\left\vert \Delta r\right\vert=0.2$ at the resonance frequency *ω*_r_/2*π* = 6.75 GHz.

To determine the coupling *κ*_c_ and losses *κ*_i_ and *κ*_s_ of our device, we fit the reflection response^32–35^ of the form

1 $$r=A/{A}_{0}=1-\frac{{\kappa }_{{{{{\rm{c}}}}}}}{\kappa /2-i(\omega -{\omega }_{{{{{\rm{r}}}}}})},$$

where *κ* = *κ*_c_ + *κ*_i_ + *κ*_s_ is the linewidth of the resonator. From the CB data, we obtain the bare resonator parameter values *κ*_c_/2*π* = 62 MHz and *κ*_i_/2*π* = 60 MHz with *κ*_s_ = 0. The CD case is then fitted with *κ*_s_/2*π* = 28 MHz as the only free parameter. Adding only this dissipation explains the changes in response from CB to CD. Therefore the QD conductance adds dissipation to the resonator. As our resonator with *κ*_i_ ≈ *κ*_c_ > *κ*_s_ is not fulfilling the matching conditions, the sensor QD changes the response only by $\left\vert \Delta r\right\vert=0.2$.

**Fig. 2:**
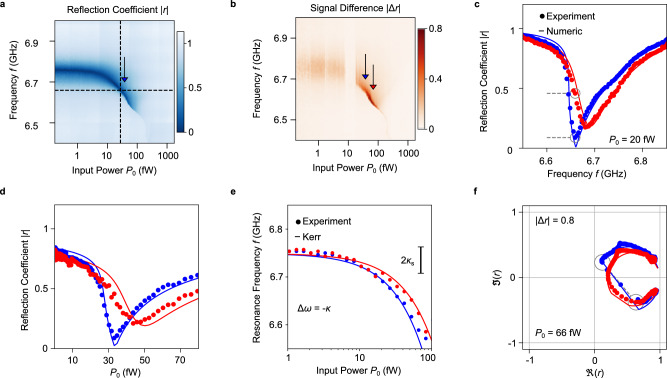
The response in the nonlinear regime. **a** The reflection coefficient ∣*r*∣ as a function of drive frequency *f* and input power *P*_0_ for CB. **b** The reflection coefficient change ∣Δ*r*∣ between CB and CD. The blue and red arrow in **a**, **b** indicate the threshold powers of Eq. (4) in CB, *P*_0_ = 43 fW, and CD, *P*_0_ = 84 fW, respectively. **c**, **d** Frequency & power response along the vertical/horizontal dashed lines of **a**, *P*_0_ = 20 fW / *f* = 6.659 GHz for CB (blue) and CD (red). The lines are numerical calculations made with the circuit model presented in the Supplemental Materials Section B, and using the low power response parameter values as input parameters. **e** The resonance frequency *f* as extracted from the minimum amplitude response of the experimental data (dots) and as predicted by the Kerr shift (lines). The solid bar shows the predicted frequency shift of Δ*ω* = 2*κ*_s_. **f** The resonator response with *P*_0_ = 66 fW shown in the IQ plane. A signal of ∣Δ*r*∣ = 0.8 is found between the CB and CD cases at frequency *f* = 6.593 GHz, indicated by the open circles and the dashed line highlighting the highest ∣Δ*r*∣ case with fixed *P*_0_ and *f*.

Next we consider the nonlinear regime of the resonator. For this, Fig. 2a repeats the measurement of Fig. 1d for different input powers *P*_0_. At *P*_0_ < 1 fW, the response is that of Fig. 1d and does not depend on *P*_0_. This is the linear response regime. At *P*_0_ > 1 fW, the resonator mode shifts to lower frequencies, and eventually vanishes. This is due to the cosine-shaped potential of the Josephson junctions, which leads to nonlinear inductance at a large input power^26,27,36,37^. The signal difference $\left\vert \Delta r\right\vert$ between the CB and CD cases is shown in Fig. 2b. Here, we see again that $\left\vert \Delta r\right\vert=0.2$ in the linear response regime, which increases to the near-unity value of $\left\vert \Delta r\right\vert=0.8$ in the nonlinear regime.

Figure 2c, d show line cuts along the dashed lines of Fig. 2a for CB and CD. Here, the response is no longer Lorentzian, but displays a steep slope on the low-frequency side arising from bifurcation in the nonlinear resonator^37–39^. Furthermore, making the QD conducting shifts the response in frequency. This is in stark contrast to the linear response of Fig. 1d, where the shift is vanishingly small. As the frequency shift moves the resonator response, the readout signal between the two states is much larger as indicated by the dashed lines in Fig. 2c, despite not satisfying the matching requirement.

To model the response theoretically, we calculate the reflection coefficient *r* of the circuit in Fig. 1b. We do this using the semiclassical Josephson equations $I={I}_{0}\sin \phi$ and $V=\frac{\hslash N}{2e}\frac{\partial \phi }{\partial t}$, for the current *I* through, and voltage *V* across the JJ array, with *ϕ* the difference in the superconducting phase across a single junction. We solve *ϕ*(*t*) using a harmonic balance method up to the first fundamental mode of the resonator for a given sinusoidal input voltage drive, with the approximation that $\sin (\phi )=\phi -{\phi }^{3}/6$, see supplemental material for details. The result of the numerical calculations are shown as the solid lines in Fig. 2c, d with the above parameter values determined in the linear response regime together with the resistance of a single SQUID *R*_J_ = 1.5 kΩ. This resistance was measured at room temperature to *R* = 1.25 kΩ, and adjusted to account for an ≈20% increase at low temperatures^40^. Together with the known number of junctions in the array as a final input parameter, this leaves no free variables in the numerical calculations apart from a 20 % (0.8 dB) correction to the input power. This correction was made to the RF calibration based on the onset of the non-linearities, and is within the typical uncertainty of ~1 dB of the microwave setups. As *κ*_s_ is the only difference between the CB and CD calculation, the theory model confirms that the nonlinear resonator turns the small dissipative response of the QD in the linear regime to a much stronger response via the induced frequency shift in Fig. 2c.

We can further quantify the onset of the nonlinear regime and the frequency shift analytically by considering the Kerr term^41^ of the resonator *E*_K_ = −*E*_C_/*N*^2^. This term leads to a resonance frequency shift

2 $$\frac{{\omega }_{{{{{\rm{K}}}}}}}{{\omega }_{{{{{\rm{r}}}}}}}=\frac{{E}_{K}n}{\hslash {\omega }_{r}}=-\frac{{E}_{{{{{\rm{C}}}}}}}{\hslash {\omega }_{{{{{\rm{r}}}}}}{N}^{2}}n=-\frac{4\pi {Z}_{{{{{\rm{r}}}}}}{\kappa }_{{{{{\rm{c}}}}}}}{{R}_{{{{{\rm{Q}}}}}}{N}^{2}{\kappa }^{2}}\frac{{P}_{0}}{\hslash {\omega }_{{{{{\rm{r}}}}}}},$$

where *E*_C_ = *e*^2^/2*C*_r_ is the charging energy of the resonator island, *R*_Q_ = *h*/*e*^2^ the resistance quantum, *n* = (4*κ*_c_*P*_0_)/(*ℏω*_*r*_*κ*^2^) the number of photons in the microwave cavity^42^, ${Z}_{{{{{\rm{r}}}}}}=\sqrt{L/{C}_{{{{{\rm{r}}}}}}}$ the characteristic impedance of the resonator and *N* the number of junctions/SQUIDs in the array. In Fig. 2e, we plot the predicted resonator frequency *ω* = *ω*_*r*_ + *ω*_K_, and find a good agreement with the experimental data. Here, the resonance frequency of the experimental data is determined from the minimum of the reflected signal.

To quantify the sensor response, we consider the resonator frequency change Δ*ω*_K_ that results from the sensor loss *κ*_s_. From Eq. (2), the resulting frequency change is

3 $$\Delta {\omega }_{{{{{\rm{K}}}}}}/{\omega }_{{{{{\rm{r}}}}}}=\frac{4\pi {Z}_{{{{{\rm{r}}}}}}{\kappa }_{{{{{\rm{c}}}}}}}{{R}_{Q}{N}^{2}{\kappa }_{{{{{\rm{CB}}}}}}^{2}}\frac{{P}_{0}}{\hslash {\omega }_{r}}\left(1-\frac{{\kappa }_{{{{{\rm{CB}}}}}}^{2}}{{({\kappa }_{{{{{\rm{CB}}}}}}+{\kappa }_{{{{{\rm{s}}}}}})}^{2}}\right),$$

where *κ*_CB_ = *κ*_c_ + *κ*_i_. To simplify the consideration further, we assume the sensor to contribute only a small addition to the resonator dissipation *κ*_s_ ≪ *κ*_CB_. Also, we consider the non-linear response regime at *ω*_K_ = −*κ* with a corresponding threshold power of

4 $${P}_{0}=\frac{\hslash {R}_{{{{{\rm{Q}}}}}}{N}^{2}{\kappa }^{3}}{4\pi {Z}_{{{{{\rm{r}}}}}}{\kappa }_{{{{{\rm{c}}}}}}},$$

derived in section D of the supplemental materials. Under this condition, the frequency shift is comparable to the linewidth, making it prominent in the response, while just reaching the bifurcation regime^38^, our resonator achieves this condition with *n* ≈ 30 photons in the resonator. With these, we obtain Δ*ω*_K_ ≈ 2*κ*_*s*_. The black vertical bar in Fig. 2e indicates this shift and matches well with the frequency shift between CB and CD.

**Fig. 3:**
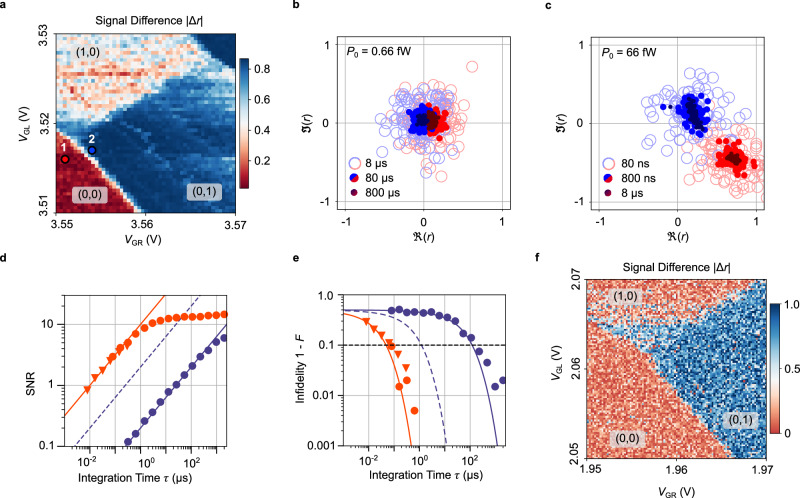
Fidelity and signal-to-noise ratio. **a** Charge stability diagram of the DQD, measured using the nonlinear detector with an integration time *τ* = 2 ms per point. Three of the visible charge configurations on the DQD are labeled as (0,0), (1,0) and (0,1). The points 1 and 2 indicate the gate voltages used for the subsequent measurements. **b**,** c** Scatter plot of the detector signal in the linear and nonlinear response regime with the DQD tuned to point 1 (red data) and point 2 (blue data). **d**,** e** Calculated signal-to-noise ratio (SNR) and fidelity as a function of the integration time *τ* for the linear (purple circles) and nonlinear regime (orange circles). For the orange triangles the room temperature setup was set for a bandwidth of 200 MHz while the other data was measured with a bandwidth of 20 MHz. The lines are fits to the data as described in the main text. The horizontal dashed line indicates a fidelity of 0.9. **f** A charge stability diagram measured with an integration time *τ* = 80 ns per point at lower DQD gate voltages of *V*_GR_ = 1.96 V and *V*_GL_ = 2.06 V. Therefore this plot presents a measurement at another charge configuration. The electron numbers indicated in the figure are with respect to this other configuration in the bottom left state. The ∣*Δr*∣ values in panels a and f are determined with respect to the bottom left corner value.

Next, we consider the readout speed for measuring a charge state of a QD system. To do this we use the input power *P*_0_ = 66 fW yielding the maximum signal ∣Δ*r*∣ = 0.8 of Fig. 2b. The corresponding response in the complex plane is shown in Fig. 2f with qualitatively different response as compared to the linear case in Fig. 1e. The measured QD system is a double quantum dot (DQD) residing in the same nanowire structure as the detector, see Fig. 1a. It couples capacitively to the QD sensor via a metallic coupler. Figure 3a presents the detector response as a function of the voltages *V*_GR_ and *V*_GL_ applied to the plunger gate electrodes of the DQD. The sensor is tuned into CD for the charge state (0,0) of the DQD. The numbers here indicate additional electrons in the DQD relative to the electron occupancy of the DQD in the botton left state. Increasing *V*_GR_ adds an electron to the right quantum dot, making the DQD switch to the state (0,1). This turns off the sensor resulting in the near unity increase of ∣Δ*r*∣ = 0.8 in the reflection signal as we see in the figure. The additional triangular region around the triple point of the charge states is likely caused either by a DQD bias voltage offset or a back action effect.

Figure 3b, c presents scatter plots of the detector signal measured repeatedly for the two charge states at the points 1 and 2 indicated in Fig. 3a. The data in the linear regime of Fig. 3b show that an integration time *τ* = 800 μs is needed to resolve the two charge states. The non-linear readout in Fig. 3c works significantly faster. The charge state can be resolved even with the smallest integration time of *τ* = 80 ns. This speed improvement arises partially from the larger readout signal used with the larger input power *P*_0_ and partially from the increased sensitivity of the nonlinear operation. To distinguish these effects from each other, we consider the fidelity *F* and signal-to-noise ratio (SNR) presented in Fig. 3d, e. Here, the fidelity is determined as the fraction of the measured data points that fall on the correct side of the discrimination line set halfway between the two charge states^43,44^. We see that in the linear case (dark-blue data) a high fidelity of 0.9, corresponding to a SNR ≈ 3, is achieved for *τ* = 200 μs. In the nonlinear case (bright-red data), the same condition is obtained already at *τ* = 80 ns. The solid lines are determined as ${{{{\rm{SNR}}}}}=\sqrt{\tau /{\tau }_{0}}$ and $F=1-1/(1+{e}^{\sqrt{\tau /{\tau }_{0}}})$, where *τ*_0_ = 25 μs (10 ns) is the integration time required for SNR = 1 in the linear (nonlinear) case. The linear case is measured at 100 times lower power corresponding to 10 times lower reflection signal. To account for this, we plot the linear response curves shifted to the corresponding 10 times higher signal level as the dashed lines in Fig. 3d, e. This signal difference explains two orders of magnitude of the readout speed difference. The nonlinear case still outperforms the linear one further by more than an order of magnitude in speed thanks to the dispersive shift. Finally, in Fig. 3f, we illustrate the fast readout by repeating the measurement of Fig. 3a at a different charge configuration with *τ* = 80 ns per point. This measurement shows clearly that both the (0, 0) ↔ (1, 0) and (0, 1) ↔ (1, 0) transitions appear with good contrast at this measurement speed.

## Discussion

Increasing the output signal amplitude *A* would allow for decreasing the fastest readout time *τ* = 80 ns of Fig. 3 further. A larger *A* is achieved by designing the device such that the non-linear regime condition *ω*_K_ = −*κ* takes place at larger input power *P*_0_. According to Eq. (2), increasing the number of junctions *N*, the onset power *P*_0_ and correspondingly the readout speed *τ* increases proportionally to *N*^2^. The larger drive power *P*_0_ also results in a larger voltage amplitude *V* in the resonator $V={\left(4{\kappa }_{{{{{\rm{c}}}}}}{\omega }_{{{{{\rm{r}}}}}}{Z}_{{{{{\rm{r}}}}}}{P}_{0}/{\kappa }^{2}\right)}^{1/2}$^45^. With the *τ* = 80 ns readout, we have *V* = 80*μ*V. This is comparable to the typical linewidth of QD sensors in the 100 μeV-range^15,17,32,46^. When the microwave amplitude exceeds the sensor linewidth, *κ*_s_ reduces^32,46,47^ leading to a contrast reduction between CB and CD. In order to increase the input power, one therefore has to keep the voltage amplitude low by either increasing the linewidth *κ* of the resonator or by reducing the characteristic impedance *Z*_r_ of the device.

Interestingly, our device scheme has a third option in form of an additional protection scheme against the microwave signal *V*: First, in CB, the usual protection scheme where the QD is non-conducting^22,23^ is obtained for our device. When the sensor quantum dot is in CB, the electron tunneling and its stochastic fluctuations are suppressed. This lowers the detector back action for this charge state. In addition, when the device is at the other charge state with the QD conducting, the resonance frequency of the cavity is shifted away from the frequency of the input signal, see the gray circles in Fig. 2c highlighting that the CD case is closer to the value ∣*r*∣ = 1 because of the frequency shift. This implies that a larger fraction of the drive signal reflects directly away from the input port instead of entering to the resonator and causing e.g. dissipative heating^15^.

Therefore, also the second charge state is protected from the readout signal. This may therefore protect the QD sensor against the microwave broadening as well as reducing the other back-action effects, such as heating^15^, sensor-induced dephasing^24^ and photo-assisted tunneling^48^. Studying the effectiveness of this protection scheme is beyond the scope of this study, and to be investigated further in future works.

As our detector scheme does not need to follow the matching requirement, it opens up new possibilities to design the charge detector. The response time *τ*_QD_ of the QD charge detector is set by the *RC*_*Σ*_ time constant where *R* = 1/*G* and *C*_*Σ*_ is the total capacitance of the quantum dot. Our device has *G* = 0.4 μS and *C*_*Σ*_ = 50 aF based on the charging energy *E*_ch_ = *e*^2^/2*C*_*Σ*_ = 1.5 meV seen in Fig. 1c. This yields an extremely fast response rate of 1/*τ*_QD_ = *G*/*C*_QD_ = 8 GHz, typical for QDs. However, in the linear response regime, the much slower coupling rate *κ*_s_ = *G*/*C*_r_ needs to be matched with the resonator coupling *κ*_c_. The overall response time of the detection speed is thus limited to the resonator linewidth taking place at this same slower timescale. The resonator total capacitance *C*_r_ is ~3 orders of magnitude larger than *C*_*Σ*_, slowing down the response time by the same fraction, typical for the various charge detection realizations^49–56^. Since the non-linear detection removes the matching requirement, a much larger *κ*_c_ can be used without suppressing the signal. This enables to decrease the resonator response timescale 1/*κ*, bringing it closer to the response time of the quantum dot *τ*_QD_ and increasing the response speed correspondingly. In the numerical calculations shown in Supplementary Fig. 5 in the supplemental materials, we increase *κ*_c_ such that *κ*/*κ*_s_ = 10, while still maintaining the near-unity signal without entering the bifurcation regime. We therefore predict that at least an order of magnitude increase in the resonator response speed is achievable. However, as *κ*_c_/*κ*_s_ increases, the frequency shift Δ*ω*_K_ becomes small compared to the linewidth. The decreasing frequency shift eventually leads to a decrease of the reflection signal ∣Δ*r*∣, setting a limit to *κ*_c_/*κ*_*s*_. In addition, the nonlinearities may decrease the response speed of the resonator, see Section D in the Supplemental materials for details. Further experiments are therefore needed for testing the achievable speed increase.

To conclude, we showed that a nonlinear resonator turns the dissipative response of a charge sensor into a frequency shift of the readout resonator. This effect allows a near unity signal strength for charge readout without having to satisfy the usual matching requirement in the resonator. As a result, we achieved an order of magnitude increase in the charge readout speed. Avoiding the matching requirement opens up an additional protection scheme against back-action effects and to make faster charge detectors approaching RC time constant of the QD sensor in the 1 ns regime.

## Methods

### Device fabrication

The device is made with 6 lithography steps on top of a high-resistivity *ρ* > 10 k*Ω* Si wafer with 200 nm thermally grown oxide. The lithography steps are:

1. EBL defined alignment markers and contact pads made with lift-off process and e-beam evaporation of 5 nm Ti + 45 nm Au.
2. UVL defined feed-lines for DC and RF signals made with lift-off process and e-beam evaporation of 5 nm Ti and 95 nm Al.
3. UVL-defined shunting capacitance made with a lift-off process. First, a 30 nm thick AlOx layer was grown, and then a 100 nm thick Al film e-beam evaporated. Following this process step, nanowires were manually transferred, and the barriers were located as in ref. ^57^.
4. EBL defined window over the nanowire for etching the GaSb shell, etching for 5 min in 2.38% TMAH.
5. EBL defined nanowire contacts with lift-off process. After development, the native oxide of InAs was removed with 1:10 HF BOE. Etch time was 5 s followed by rinsing in water and loading immediately to the evaporator. Then 5 nm Ti and 135 nm Al was deposited with e-beam evaporator.
6. EBL defined Niemeyer-Dolan bridge for the resonator array. The resist stack consisted of 3 layers of MMA(8.5)MAA copolymer, 9% solids in Ethyl Lactate, spin-coated at 4000 rpm. The top layer is 950,000 molecular weight PMMA, 6% solids in Anisole, spin coated with 5000 rpm. The Josephson junctions and the rest of the resonator structure was formed with a two-angle evaporation of Al at ±30^∘^, with an intermediate oxidation step determining the resistance of the resulting Josephson junctions.

The device was then wire bonded to the measurement PCB and measured at room temperature to determine the resistance of the Josephson junctions and quantum dots.

### Measurement setup

The measurements were performed in a dilution refrigerator at a base temperature of 10 mK. The measurement setup was identical to ref. ^58^ with the following differences: The 80 MHz low pass filter after the mixer was removed, and an amplifier with 200 MHz bandwidth (Femto HVA-200M-40-B with AC coupling and 20 dB gain) was used to allow for a larger bandwidth in the measurement. The signal was then digitized with an oscilloscope and the in-phase and quadrature components were determined. An intermediate frequency (IF) of 12.5 MHz was used for the data presented in Figs. 1, 2, and the 20 MHz data of Fig. 3. The oscilloscope sampling rate was set to 50 MS/s and the analog bandwidth to 20 MHz. For the 200 MHz data in Fig. 3, the intermediate frequency was increased 125 MHz, the sampling rate to 500 MS/s with full analog bandwidth of the oscilloscope. Then the IF amplifier Femto HVA-200M-40-B was defining the overall analog bandwidth of the setup to 200 MHz. Note that the intermediate frequencies here were chosen to be approximately a factor of two lower than the analog bandwidth. This lowers the time resolution by a corresponding amount as one needs to have at least one oscillation period of the IF signal in order to determine the response. Using this lower intermediate frequency was done in order to avoid excessive signal distortions such as signal compression and stronger frequency dependence in the attenuation that are induced if the IF signal were precisely at the bandwidth specifications of the analog components.

## Supplementary information

Supplementary Information

Transparent Peer Review file

## Data Availability

The data generated in this study have been deposited in the Zenodo database under accession code 15672394^59^.

## References

[1] Schoelkopf, R. J., Wahlgren, P., Kozhevnikov, A. A., Delsing, P. & Prober, D. E. The radio-frequency single-electron transistor (RF-SET): a fast and ultrasensitive electrometer. *Science* **280**, 1238 (1998). 9596572 10.1126/science.280.5367.1238

[2] Pekola, J. P. et al. Single-electron current sources: toward a refined definition of the ampere. *Rev. Mod. Phys.* **85**, 1421 (2013).

[3] Vigneau, F. et al. Probing quantum devices with radio-frequency reflectometry. *Appl. Phys. Rev.* **10**, 021305 (2023).

[4] Mi, X. et al. A coherent spin-photon interface in silicon. *Nature* **555**, 599 (2018). 29443961 10.1038/nature25769

[5] Zheng, G. et al. Rapid gate-based spin read-out in silicon using an on-chip resonator. *Nat. Nanotech.* **14**, 742 (2019). 10.1038/s41565-019-0488-9 31285611

[6] Chatterjee, A. et al. Semiconductor qubits in practice. *Nat. Rev. Phys.* **3**, 157 (2021).

[7] Oakes, G. A. et al. Fast high-fidelity single-shot readout of spins in silicon using a single-electron box. *Phys. Rev. X* **13**, 011023 (2023).

[8] Burkard, G., Ladd, T. D., Pan, A., Nichol, J. M. & Petta, J. R. Semiconductor spin qubits. *Rev. Mod. Phys.* **95**, 025003 (2023).

[9] Keller, M. W., Eichenberger, A. L., Martinis, J. M. & Zimmerman, N. M. A capacitance standard based on counting electrons. *Science* **285**, 1706 (1999). 10481001 10.1126/science.285.5434.1706

[10] Gustavsson, S. et al. Electron counting in quantum dots. *Surf. Sci. Rep.* **64**, 191 (2009).

[11] Albert, M., Flindt, C. & Büttiker, M. Distributions of waiting times of dynamic single-electron emitters. *Phys. Rev. Lett.* **107**, 086805 (2011). 21929192 10.1103/PhysRevLett.107.086805

[12] Küng, B. et al. Irreversibility on the level of single-electron tunneling. *Phys. Rev. X* **2**, 011001 (2012).

[13] Wagner, T. et al. Strong suppression of shot noise in a feedback-controlled single-electron transistor. *Nat. Nanotech.* **12**, 218 (2017). 10.1038/nnano.2016.225 27819692

[14] Tanttu, T. et al. Controlling spin-orbit interactions in silicon quantum dots using magnetic field direction. *Phys. Rev. X* **9**, 021028 (2019).

[15] Keith, D. et al. Single-shot spin readout in semiconductors near the shot-noise sensitivity limit. *Phys. Rev. X* **9**, 041003 (2019).

[16] Turin, V. O. & Korotkov, A. N. Analysis of the radio-frequency single-electron transistor with large quality factor. *Appl. Phys. Lett.* **83**, 2898 (2003).

[17] Ares, N. et al. Sensitive radio-frequency measurements of a quantum dot by tuning to perfect impedance matching. *Phys. Rev. Appl.* **5**, 034011 (2016).

[18] Sillanpää, M. A., Roschier, L. & Hakonen, P. J. Inductive single-electron transistor. *Phys. Rev. Lett.* **93**, 066805 (2004). 15323652 10.1103/PhysRevLett.93.066805

[19] Thyagarajan, B. et al. Fast high-fidelity charge readout by operating a cavity-embedded cooper-pair transistor in the kerr bistable regime. *Phys. Rev. Appl.* **21**, 014064 (2024).

[20] Wallraff, A. et al. Approaching unit visibility for control of a superconducting qubit with dispersive readout. *Phys. Rev. Lett.* **95**, 060501 (2005). 16090931 10.1103/PhysRevLett.95.060501

[21] Lupaşcu, A., Driessen, E. F. C., Roschier, L., Harmans, C. J. P. M. & Mooij, J. E. High-contrast dispersive readout of a superconducting flux qubit using a nonlinear resonator. *Phys. Rev. Lett.* **96**, 127003 (2006). 16605947 10.1103/PhysRevLett.96.127003

[22] Morello, A. et al. Single-shot readout of an electron spin in silicon. *Nature* **467**, 687 (2010). 20877281 10.1038/nature09392

[23] Park, J. et al. Passive and active suppression of transduced noise in silicon spin qubits. *Nat. Commun.* **16**, 78 (2025). 39747837 10.1038/s41467-024-55338-z PMC11696227

[24] Haldar, S. et al. Coherence of an electronic two-level system under continuous charge sensing by a quantum dot detector. *Phys. Rev. Lett.* **134**, 023601 (2025). 39913833 10.1103/PhysRevLett.134.023601

[25] Hermon, Z., Ben-Jacob, E. & Schön, G. Charge solitons in one-dimensional arrays of serially coupledJosephson junctions. *Phys. Rev. B* **54**, 1234 (1996). 10.1103/physrevb.54.1234 9985395

[26] Castellanos-Beltran, M. A. & Lehnert, K. W. Widely tunable parametric amplifier based on a superconducting quantum interference device array resonator. *Appl. Phys. Lett.* **91**, 083509 (2007).

[27] Bell, M. T., Sadovskyy, I. A., Ioffe, L. B., Kitaev, A. Y. & Gershenson, M. E. Quantum superinductor with tunable nonlinearity. *Phys. Rev. Lett.* **109**, 137003 (2012). 23030113 10.1103/PhysRevLett.109.137003

[28] Altimiras, C. et al. Tunable microwave impedance matching to a high impedance source using aJosephson metamaterial. *Appl. Phys. Lett.* **103**, 212601 (2013).

[29] Stockklauser, A. et al. Strong coupling cavityQED with gate-defined double quantum dots enabled by a high impedance resonator. *Phys. Rev. X* **7**, 011030 (2017).

[30] Scarlino, P. et al. All-microwave control and dispersive readout of gate-defined quantum dot qubits in circuit quantum electrodynamics. *Phys. Rev. Lett.* **122**, 206802 (2019). 31172788 10.1103/PhysRevLett.122.206802

[31] Harabula, M.-C. et al. Measuring a quantum dot with an impedance-matching on-chip superconducting*L**C* resonator at gigahertz frequencies. *Phys. Rev. Appl.* **8**, 054006 (2017).

[32] Havir, H. et al. Quantum dot source-drain transport response at microwave frequencies. *Phys. Rev. B* **108**, 205417 (2023).

[33] Göppl, M. et al. Coplanar waveguide resonators for circuit quantum electrodynamics. *J. Appl. Phys.* **104**, 113904 (2008).

[34] Probst, S., Song, F. B., Bushev, P. A., Ustinov, A. V. & Weides, M. Efficient and robust analysis of complex scattering data under noise in microwave resonators. *Rev. Sci. Instrum.* **86**, 024706 (2015). 25725869 10.1063/1.4907935

[35] Rieger, D. et al. Fano interference in microwave resonator measurements. *Phys. Rev. Appl.* **20**, 014059 (2023).

[36] Tholén, E. A. et al. Nonlinearities and parametric amplification in superconducting coplanar waveguide resonators. *Appl. Phys. Lett.* **90**, 253509 (2007).

[37] Bishop, L. S., Ginossar, E. & Girvin, S. M. Response of the strongly driven Jaynes-Cummings oscillator. *Phys. Rev. Lett.* **105**, 100505 (2010). 20867501 10.1103/PhysRevLett.105.100505

[38] Ong, F. R. et al. CircuitQED with a nonlinear resonator: AC-Stark shift and dephasing. *Phys. Rev. Lett.* **106**, 167002 (2011). 21599402 10.1103/PhysRevLett.106.167002

[39] Frasca, S. et al. NbN films with high kinetic inductance for high-quality compact superconducting resonators. *Phys. Rev. Appl.* **20**, 044021 (2023).

[40] Holmqvist, T., Meschke, M. & Pekola, J. P. Double oxidation scheme for tunnel junction fabrication. *J. Vacuum Sci. Techol. B* **26**, 28 (2008).

[41] Koch, J. et al. Charge-insensitive qubit design derived from theCooper pair box. *Phys. Rev. A* **76**, 042319 (2007).

[42] Andersson, S., Havir, H., Ranni, A., Haldar, S. & Maisi, V. F. High-impedance microwave resonators with two-photon nonlinear effects. *Nat. Commun.* **16**, 552 (2025). 39788991 10.1038/s41467-025-55860-8 PMC11718305

[43] Schaller, G., Kießlich, G. & Brandes, T. Low-dimensional detector model for full counting statistics: trajectories, back action, and fidelity. *Phys. Rev. B* **82**, 041303 (2010).

[44] Chen, L. et al. Transmon qubit readout fidelity at the threshold for quantum error correction without a quantum-limited amplifier. *npj Quantum Inf.* **9**, 26 (2023).

[45] Haldar, S. et al. Energetics of microwaves probed by double quantum dot absorption. *Phys. Rev. Lett.* **130**, 087003 (2023). 36898111 10.1103/PhysRevLett.130.087003

[46] Frey, T. et al. Characterization of a microwave frequency resonator via a nearby quantum dot. *Appl. Phys. Lett.* **98**, 262105 (2011).

[47] Cornia, S. et al. Microwave-assisted tunneling in hard-wall InAs/InP nanowire quantum dots. *Sci. Rep.* **9**, 19523 (2019). 31863018 10.1038/s41598-019-56053-2 PMC6925118

[48] Mavalankar, A. et al. Photon-assisted tunneling and charge dephasing in a carbon nanotube double quantum dot. *Phys. Rev. B* **93**, 235428 (2016).

[49] Buehler, T. M. et al. Development and operation of the twin radio frequency single electron transistor for cross-correlated charge detection. *J. Appl. Phys.* **96**, 4508 (2004).

[50] Bylander, J., Duty, T. & Delsing, P. Current measurement by real-time counting of single electrons. *Nature* **434**, 361 (2005). 15772655 10.1038/nature03375

[51] Angus, S. J., Ferguson, A. J., Dzurak, A. S. & Clark, R. G. A silicon radio-frequency single electron transistor. *Appl. Phys. Lett.* **92**, 112103 (2008).

[52] Dehollain, J. P. et al. Single-shot readout and relaxation of singlet and triplet states in exchange-coupled ^31^P electron spins in silicon. *Phys. Rev. Lett.* **112**, 236801 (2014). 24972221 10.1103/PhysRevLett.112.236801

[53] Lu, W., Ji, Z., Pfeiffer, L., West, K. W. & Rimberg, A. J. Real-time detection of electron tunnelling in a quantum dot. *Nature* **423**, 422 (2003). 12761544 10.1038/nature01642

[54] Elzerman, J. M. et al. Single-shot read-out of an individual electron spin in a quantum dot. *Nature* **430**, 431 (2004). 15269762 10.1038/nature02693

[55] Reilly, D. J., Marcus, C. M., Hanson, M. P. & Gossard, A. C. Fast single-charge sensing with aRF quantum point contact. *Appl. Phys. Lett.* **91**, 162101 (2007).

[56] Barthel, C. et al. Fast sensing of double-dot charge arrangement and spin state with a radio-frequency sensor quantum dot. *Phys. Rev. B* **81**, 161308 (2010).

[57] Barker, D. et al. Experimental verification of the work fluctuation-dissipation relation for information-to-work conversion. *Phys. Rev. Lett.* **128**, 040602 (2022). 35148140 10.1103/PhysRevLett.128.040602

[58] Haldar, S. et al. Continuous microwave photon counting by semiconductor-superconductor hybrids. *Phys. Rev. Lett.* **133**, 217001 (2024). 39642513 10.1103/PhysRevLett.133.217001

[59] Havir, H. Source data for “near-unity charge readout in a nonlinear resonator without matching”. Preprint at 10.5281/zenodo.15672394 (2025).

