# Supplementary material for: Near-unity charge readout signal in a nonlinear resonator without matching the sensor dissipation
Source: Nat Commun. 2026 Jul 2;17:5781. doi: 10.1038/s41467-026-75082-w (PMC13328590; doi:10.1038/s41467-026-75082-w)
Supplement: Supplementary file 1 — Supplementary Information [file 41467_2026_75082_MOESM1_ESM.pdf]

Supplementary Materials to Near-Unity Charge Readout Signal in a Nonlinear Resonator without Matching the Sensor Dissipation

Harald Havir [1], Andrea Cicovic,[1] Pierre Glidic,[1,2] Subhomoy Haldar,[1,3] Sebastian Lehmann,[1] Kimberly A. Dick,[1,4] and Ville F. Maisi [1,*]

[1] NanoLund and Solid State Physics, Lund University, Box 118, 22100 Lund, Sweden
[2] Université Lyon 1, CNRS, Institut Lumière Matière, UMR5306, F-69100, Villeurbanne, France
[3] Department of Physics, Indian Institute of Technology Kanpur, Uttar Pradesh 208016, India
[4] Center for Analysis and Synthesis, Lund University, Box 124, 22100 Lund, Sweden

A. QD sensor characterization

Supplementary Figure 1 presents the sensor QD conductance G and the sensor dissipation κ_s as a function of the gate voltage V_G for the operation point studied in Figs. 1 - 3a of the main article. The conductance was determined from the slope of the IV curve over a 20 μV bias range around the zero bias voltage. The sensor dissipation κ_s was determined with the same procedure as for the red data set in Fig. 1d.

Supplementary Figure 2 presents characterization data that we use for determining the capacitive coupling strength of the DQD to the charge sensor QD. Supplementary Figure 2 presents a charge stability diagram of the DQD measured by probing the direct current I_{SD} of the sensor QD with a fixed voltage bias $V_b = 0.4$ mV. This method with a finite bias and measuring the direct current I_{SD} is chosen here to have a wider gate voltage span with charge sensitivity, see panel **c** where the sensitivity range with the corresponding charge states $(0,0)$, $(0,1)$ and $(1,0)$ are indicated, and cf. to Supplementary Fig. 1. The measurement here was made so that the QD sensor was first tuned to the point indicated with "$(0,0)$" in panel c while the DQD gate voltages were set to $V_{GL} = 0.91$ V and $V_{GR} = 0.88$ V. Then the measurement of panel **a** was made where these gate voltages are swept. When the DQD occupancy changes, the current I_{SD} increases as indicated in panel **c** for the three charge states $(0,0)$, $(0,1)$ and $(1,0)$. Supplementary Fig. 2 presents linecuts along the lines indicated in panel **a**. These line cuts show the occupancy changes $(0,0) \leftrightarrow (0,1)$, $(0,0) \leftrightarrow (1,0)$ and $(0,1) \leftrightarrow (1,0)$.

To quantify the capacitive coupling between the DQD and the QD sensor, we determine how large fraction of the added electron "gates" the QD sensor. If the capacitive coupling would be very strong, the full charge of the added electron, e.g. for the $(0,0) \rightarrow (0,1)$ transition, would appear on the sensor QD, and the sensor QD would be shifted by a full Coulomb oscillation period in gate voltage V_G. The capacitive coupling is however much weaker, and as we can see from Supplementary Fig. 2**c**, the shift in gate voltage is $\Delta V_G = 1.1$ mV between the charge states $(0,0)$ and $(0,1)$. As one Coulomb oscillation period is $\Delta V_G = 13$ mV, the $(0,0) \rightarrow (0,1)$ transition induces a 2 mV/(13 mV/e) = 0.08e charge change at the sensor QD. Similarly for the $(0,0) \rightarrow (1,0)$ transition we obtain $\Delta V_G = 2$ mV leading to 0.15e, and for the $(0,1) \rightarrow (1,0)$ transition $\Delta V_G = 0.9$ mV leading to 0.07e.

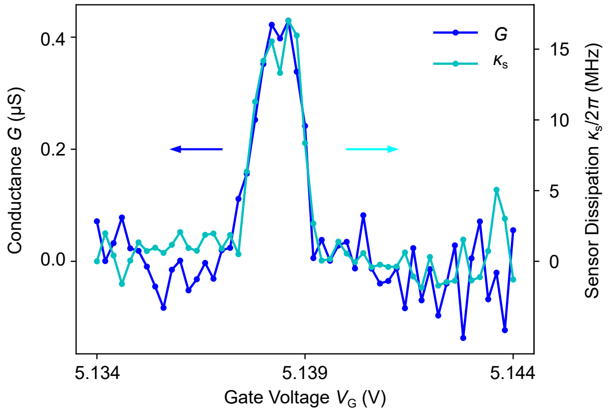

Supplementary Figure 1. Quantum Dot Conductance and Dissipation. The sensor quantum dot conductance G and dissipation κ_s as a function of the gate voltage V_G.

B. Circuit Model

For modeling the detector response, we solve the voltages and currents in the circuit of Fig. 1b under the sinusoidal input signal used in the experiment. The RF sinusoidal input signal with power P_0 and amplitude A_0 entering via a $Z_0 = 50\,\Omega$ transmission line is equivalent of a voltage supply with voltage $V_{in}(t) = V_0\left(e^{i\omega t} + e^{-i\omega t}\right)/2$, and internal resistance of Z_0, where $V_0 = |A_0|$. The amplitude V_0 connects to the input power as $P_0 = (V_0/2\sqrt{2})^2/Z_0$. Here the $\sqrt{2}$ accounts for the difference between the RMS value of the voltage and its amplitude, and the factor of two for considering the power P_0 at the output after the internal resistance. Here it is important to make sure that the input drive $V_{in}(t)$ is properly real-valued. Using e.g. $V_{in}(t) = V_0 e^{i\omega t}$, which is often done for linear circuits does not work for the non-linear case. This because taking a real value of this complex-valued solution is not anymore a solution for

* ville.maisi@ftf.lth.se

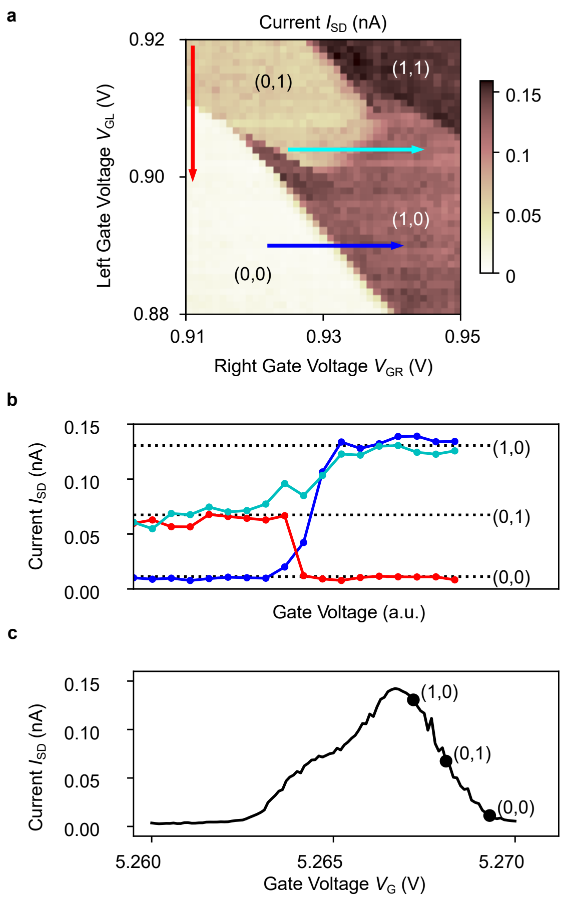

Supplementary Figure 2. Sensor coupling to DQD charge states. a The sensor quantum dot current $I_{\rm SD}$ as a function of the double dot gate voltage $V_{\rm GL}$ and $V_{\rm GR}$ for one more charge configuration. Note that the DQD gate voltages here differ from the settings used in the main article. The indicated charge occupations $(0,0)$, $(0,1)$, $(1,0)$, $(1,1)$ here indicate again the number of added electrons in relation to the state in the bottom left corner. The QD voltage bias is $V_b = 0.4$ mV. **b** Linecuts made as indicated in panel **a**. **c** The QD sensor response as a function of the gate voltage $V_{\rm G}$ for the applied bias voltage $V_b = 0.4$ mV. The dots indicate the three charge states of panel **a**.

the non-linear equation. That procedure works only for a linear differential equation where taking the real value yields directly a real-valued solution.

For the Josephson junctions, we use the semiclassical Josephson relations

$$\begin{cases} I = I_0 \sin\phi \\ V = \dfrac{\hbar N}{2e}\dfrac{\partial \phi}{\partial t}, \end{cases} \tag{1}$$

for the current I and voltage V across N identical junctions in series. The Kirchhoff's rules for the components in Fig. 1b (conservation of current in the nodes, and voltages adding up to zero in the loops), yield then the equation of motion

$$\frac{\partial^2 \phi}{\partial t^2} + (\kappa_{\rm s} + \kappa_{\rm c})\frac{\partial \phi}{\partial t} + \omega_{\rm r}^2 \sin\phi = c_{\rm c}\omega_{\rm r}\frac{\partial v_{\rm in}(t)}{\partial t}, \tag{2}$$

for the phase ϕ across the array. Here $\omega_{\rm r} = 1/\sqrt{LC_{\rm r}}$ is the resonance frequency in the low power linear regime with inductance $L = \frac{\hbar N}{2eI_0}$, and capacitance $C_{\rm r} = C + C_{\rm c}\,(1 + Z_0 G)$, and $c_{\rm c} = C_{\rm c}/C_{\rm r}$. The QD loss term is $\kappa_{\rm s} = G/C_{\rm r}$, i.e. the RC time constant arising from the conductance G. Note here that the finite frequency conductance usually differs from the low-frequency one [1], leading to a difference between the low-frequency and high-frequency dissipation. The loss via the input port takes the usual [2, 3] form $\kappa_{\rm c} = \frac{Z_0 C_1^2 \omega_{\rm r}^2}{C_{\rm r}}\,(1 + Z_0 G)$, when combining the terms $Z_0 C_1 \omega_{\rm r}^2 \cos\phi\,\frac{\partial\phi}{\partial t}$ and $\frac{Z_0 C C_{\rm c}}{C_{\rm r}}\frac{\partial^3\phi}{\partial t^3}$. Here we approximated $\cos\phi = 1$ to the leading order in ϕ, and consider a solution of the form $\phi = \phi_1 e^{i\omega t} + \phi_1^* e^{-i\omega t}$. This ansatz allows to approximate $\frac{\partial^3\phi}{\partial t^3} = -\omega^2\frac{\partial\phi}{\partial t} = -\omega_{\rm r}^2\frac{\partial\phi}{\partial t}$ close to the resonator frequency $\omega_{\rm r}$ to obtain the above result for $\kappa_{\rm c}$. The right hand side of Eq. (2) is the drive term with normalization $v_{\rm in}(t) = \frac{2e}{N\hbar\omega_{\rm r}} V_{\rm in}(t)$.

Next with the Taylor expansion $\sin\phi \approx \phi - \phi^3/6$, Eq. (2) has the form of a Duffing oscillator. The harmonic balance method yields then the equation

$$\phi_1 = \frac{i\omega c_{\rm c} v_0/2\omega_{\rm r}}{1 - \omega^2/\omega_{\rm r}^2 + i\omega\kappa/\omega_{\rm r}^2 - |\phi_1|^2/2}, \tag{3}$$

for the amplitude ϕ_1 of the phase response when solving the equation for the fundamental mode at ω and neglecting the higher frequency components. Equation (3) is easily solved with fixed point iteration to obtain the amplitude ϕ_1. Here $v_0 = \frac{2e}{N\hbar\omega_{\rm r}} V_0$, is the normalized input amplitude. Then the resonator impedance Z is determined by calculating the voltage and current in front of the input capacitor $C_{\rm c}$. This results in

$$Z = \frac{v_0/2 - \omega^2 C_{\rm c} Z_0 \phi_1/\omega_{\rm r}}{i\omega C_{\rm c}(v_0/2 - i\omega\phi_1/\omega_{\rm r})}. \tag{4}$$

The reflected signal is then finally given by the standard formula

$$r = \frac{Z - Z_0}{Z + Z_0}. \tag{5}$$

The reflection coefficient r here is often denoted with Γ instead of the r. We chose to use r to avoid confusion with the usual notation in our field where Γ is often used to indicate various rates.

Supplementeray Figure **3** presents the experimental data for both CB and CD cases and their difference on the left column. The corresponding circuit model calculations are given on the right column. The parameter values of the device are summarized in Supplementary Table **1**. These are used for all the modeling.

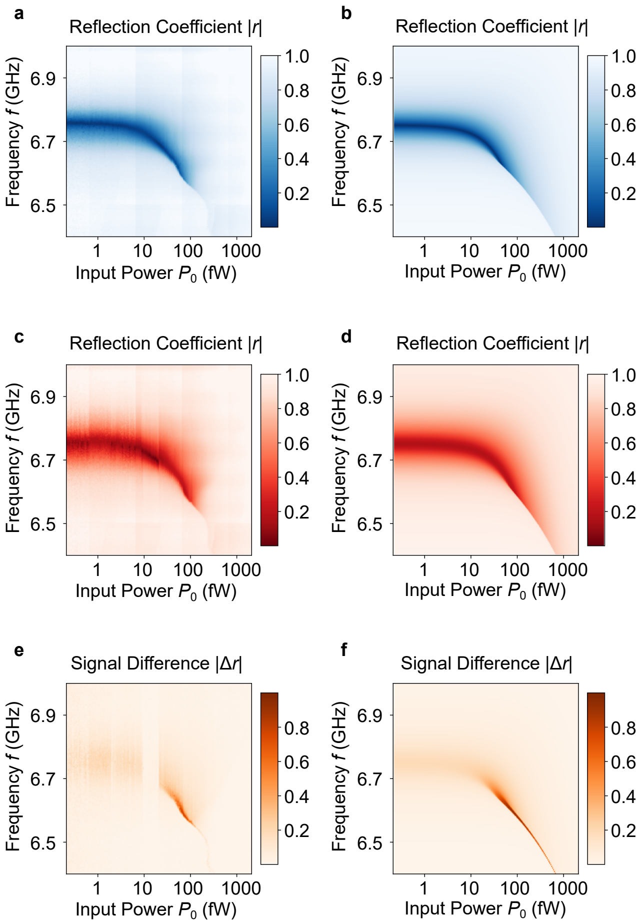

Supplementary Figure 3. **Experiment and Simulation of Nonlinear Response.** **a** Measured resonator response $|r|$ for CB as in Fig. 2 **a** of the main manuscript. **b** Corresponding circuit model result. **c** & **d** Same data as in panels **a** and **b** but now in CD. **e** & **f** the resulting signal difference $|\Delta r|$ between the CD and CB results.

C. Estimation of the resonator response speed reduction from nonlinear effects

The response time considerations of the main article are based on the response time of a linear system. The nonlinear effects may change this response time and possibly slow down the detection. We estimate this reduction by estimating how much the coupling term κ_c changes for the operation point used in Fig. 3. The circuit model yields the highest phase amplitude of $|\phi_1| = 0.1\pi$ for this considered operation point. The nonlinear effects contribute therefore at most 5 % corrections to the linear leading order terms of the equation of motion Supplementary Equation (2) via the $\sin\phi$ and $\cos\phi$ terms. The reduction of κ_c arises partly from the reduction of the used frequency ω and partly from the correction to the approximation $\cos\phi \approx 1$ made in the derivation of κ_c. Using the observed frequency shift, and the highest phase value of $\phi = |\phi_1| = 0.1\pi$ to estimate the error in the above approximation, reduces the κ_c value by 5 %. We estimate that for the considered operation point, the reduction of the response time is of similar size, and hence does not slow down the detector significantly. This assessment is further supported by Refs. 4–7 reporting the transient response in the nonlinear regime taking place in a similar timescale as in the linear response regime. Further experiments probing the response time are, however, needed for testing if this estimation is valid.

Supplementary Table 1. **Summary of the parameters and their values.**

Name	Parameter	Value
Input coupling rate	$\kappa_c/2\pi$	62 MHz
Internal losses	$\kappa_i/2\pi$	60 MHz
Sensor dissipation rate	$\kappa_s/2\pi$	28 MHz
Resonance frequency	$\omega_r/2\pi$	6.75 GHz
Number of junctions	N	13
Junction resistance	R_J	1.5 kΩ
Junction inductance	L_J	1.7 nH
Resonator total capacitance	C_r	25 fF
Coupler capacitance	C_c	10 fF
Resonator impedance	Z_r	950 Ω

D. The Kerr coefficient

The Kerr term with the coefficient E_K describes the non-linearity of a resonator. With the resonance frequency ω_r and photon creation and annihilation operators $\hat{a}^\dagger$ and $\hat{a}$, the corresponding Hamiltonian reads

$$\hat{H} = \hbar\omega_r \hat{a}^\dagger\hat{a} + \frac{E_K}{2}\hat{a}^\dagger\hat{a}^\dagger\hat{a}\hat{a}, \tag{6}$$

resulting in the energy difference between n and $n-1$ photons as

$$E_n = \hbar\omega_r + E_K(n-1). \tag{7}$$

In other words, the resonance frequency is shifted by $\omega_K = \frac{E_K}{\hbar}(n-1)$ for n photons in the resonator, relative to the lowest photon transition frequency of $E_1/\hbar = \omega_r$. For the weak non-linearity considered in this work, this shift is only significant for $n \gg 1$ allowing us to approximate $n-1 \approx n$ for the shift.

The Kerr coefficient is $E_K = -E_C$ for a single Josephson junction [3, 8–11]. For the N junction array considered in this work, the total resonator voltage V is divided across the N junctions. The photon number n in the resonator is proportional to the energy stored in the resonator, which in turn is proportional to V^2. Therefore, to reach the same voltage amplitude across a single junction in the array, the photon number needs to be N^2 times larger as compared to the single junction case. Therefore, the Kerr coefficient for the array is $E_K = -E_C/N^2$. Here it is important to note that the

"charging energy" $E_C = e^2/2C_r$ is calculated for the resonator total capacitance C_r. It is therefore the charging energy of the resonator, not that of the QD. Using the relation $\omega_r = 1/\sqrt{LC_r}$, for the resonance frequency and $Z_r = \sqrt{L/C_r}$, for the characteristic impedance of the resonator at low power limit, the Kerr coefficient becomes

$$E_K = -\hbar\omega_r \frac{\pi Z_r}{R_Q N^2}, \tag{8}$$

where $R_Q = h/e^2$, is the resistance quantum. We therefore see that the characteristic impedance Z_r and the number of junctions N are the key parameters determining the resonator non-linearity via the Kerr coefficient.

Considering further that the relation between the input power P_0 and the number of photons n in the linear resonator response regime [3, 12] is

$$n = \frac{4\kappa_c}{\kappa^2}\frac{P_0}{\hbar\omega_r}, \tag{9}$$

we obtain the frequency shift as

$$\omega_K/\omega_r = -\frac{4\pi Z_r \kappa_c}{R_Q N^2 \kappa^2}\frac{P_0}{\hbar\omega_r}. \tag{10}$$

The non-linearity becomes visible in the response when it is comparable to the resonator linewidth: $\omega_K = -\kappa$. This condition yields the threshold input power between the linear and non-linear response regime as

$$P_0 = \frac{\hbar R_Q N^2 \kappa^3}{4\pi Z_r \kappa_c}. \tag{11}$$

The frequency shift $\Delta\omega_K = 2\kappa_s$ resulting in from the sensor dissipation κ_s is obtained by using this power and Taylor expanding Supplementary Equation (10) in κ_s.

E. Bifurcation threshold

This section shows that the optimal input power for the nonlinear regime is indeed obtained at the $\omega_K \approx -\kappa$ condition. We consider the bifurcation threshold condition [13], that is, the largest input power P_0 where the resonator still has a unique amplitude solution $|\phi_1|$ for all input frequencies. We also show that the above relation as well as Supplementary Equation (11) - that was obtained with the linear resonator photon number of Supplementary Equation (9) - is correct within ~ 20 %, and obtain an analytical equation for the amplitude $|\phi_1|$ at the bifurcation threshold.

We start by rewriting Supplementary Equation (3) as

$$\left[(1-x-y/2)^2 + \alpha x\right] y = \beta x, \tag{12}$$

where $x = (\omega/\omega_r)^2$, $y = |\phi_1|^2$, $\alpha = (\kappa/\omega_r)^2$ and $\beta = (c_c v_0/2)^2$. For the coming argumentation, it is useful to define the function

$$f(x,y) = \left[(1-x-y/2)^2 + \alpha x\right] y - \beta x. \tag{13}$$

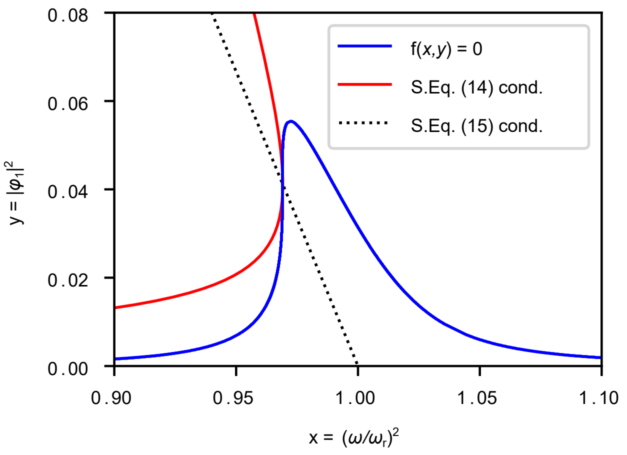

Supplementary Figure 4. Resonator response at the bifurcation threshold. The solid blue line shows the resonator phase amplitude $|\phi_1|$ as a function of the input drive frequency ω for the studied resonator with $\alpha = (\kappa/\omega_r)^2 = 3.3\cdot 10^{-4}$, and input drive value at the bifurcation threshold value of $\beta = 16\alpha^{3/2}/3\sqrt{3} = 1.8\cdot 10^{-5}$. The curve is obtained by solving numerically the condition $f(x,y) = 0$. The solid red curve plots Supplementary Eq. (14) and the dashed black line Supplementary Eq. (15). The three curves cross at the bifurcation onset point $x = 1-\sqrt{3\alpha} = 0.97$, $y = 4\sqrt{\alpha/3}$ =0.042, with $\partial f/\partial y = \partial g/\partial y = 0$ as required for the onset point determining the bifurcation threshold.

The solutions of Supplementary Equation (12) are then $f(x,y) = 0$, yielding the possible values of the oscillation amplitudes y for a given input frequency x. Supplementary Figure 4 plots these solutions as a solid blue line with the value $\alpha = 3.3\cdot 10^{-4}$ valid for the resonator in the main manuscript, and input drive of $\beta = 1.8\cdot 10^{-5}$.

The bifurcation regime takes place for large input drive $\beta = (c_c v_0/2)^2$ such that the $\omega < \omega_r$ side has multiple solutions for $|\phi_1|$. The bifurcation threshold, i.e. drive value β above which multiple solutions exist, needs to fulfill *Condition I:* The blue curve satisfying $f(x,y) = 0$ in Supplementary Figure 4 has one and only one point for which $dy/dx \to \infty$. This condition is fulfilled if and only if *Condition II:* $\partial f(x,y)/\partial y = 0$, and *Condition III:* The $\partial f(x,y)/\partial y = 0$ curve is tangential to the $f(x,y) = 0$ curve, are both satisfied at that one point. The Condition II makes sure that $dy/dx \to \infty$ is satisfied, and the Condition III with the two curves tangential makes sure that there is only one point where Condition II is satisfied. If Condition III is not fulfilled, either no solutions exists with the curves never crossing (small β) or two solutions exist as two crossing points (large β in the bifurcation regime). The values of x, y and β at that single point describe the bifurcation threshold. The multivalued amplitudes $|\phi_1|$ develop around this point for the larger input drives. Therefore, it makes sense to call this point the bifurcation threshold point.

The Condition II is easily determined to yield the equa-

tion

$$g(x,y) = (1 - x - y/2)\, y^2 - \beta x = 0. \tag{14}$$

Here we have evaluated the partial derivative $\partial f/\partial y = 0$, used the fact that $f(x,y) = 0$ for the considered point, and multiplied the resulting equation by y. The solution of Supplementary Equation (14) is shown as solid red line in Supplementary Figure 4. The Condition III, $\partial f(x,y)/\partial y = 0$ tangential to $f(x,y) = 0$, is equivalent to $\partial g/\partial y = 0$, i.e. that $g(x,y) = 0$ is tangential to $f(x,y) = 0$, when noting that the tangent of $f(x,y) = 0$ is along the y-axis for the bifurcation threshold point. With the requirement $\partial g/\partial y = 0$, we obtain thus

$$1 - x = \frac{3}{4} y, \tag{15}$$

which is presented at dashed black line in Supplementary Figure 4. Substituting Supplementary Equation (15) to Supplementary Equation (14) yields $\beta x = y^3/4$. Substituting this and Supplementary Equation (15) further to the condition $f(x,y) = 0$ results in $y/4 = \sqrt{\alpha/3}$. Here we have assumed $\alpha \ll 1$, i.e. that the linewidth is much smaller than the resonance frequency, $\kappa \ll \omega_r$, a condition typically valid for resonators.

As a summary, for a given resonator linewidth α, the bifurcation threshold takes place at the frequency x, amplitude y and input drive β given by

$$\begin{cases} 1 - x &= \sqrt{3\alpha} \\ y &= 4\sqrt{\alpha/3} \\ \beta &= 16\alpha^{3/2}/3\sqrt{3} \end{cases}. \tag{16}$$

In the non-normalized units, this bifurcation threshold point is expressed as

$$\begin{cases} \omega - \omega_r &= -\dfrac{\sqrt{3}}{2}\kappa \\ |\phi_1|^2 &= \dfrac{4\kappa}{\sqrt{3}\omega_r} \\ P_0 &= \dfrac{\hbar R_Q N^2 \kappa^3}{3\sqrt{3}\pi Z_r \kappa_c}, \end{cases}. \tag{17}$$

where a Taylor expansion $1 - x = 1 - (\omega/\omega_r)^2 = 2(\omega - \omega_r)/\omega_r + \mathcal{O}\left([(\omega - \omega_r)/\omega_r]^2\right)$ around ω_r was used for the first expression. We see that the first equation matches with the condition $\omega_K = -\kappa$ and the last one with the condition of Eq. (11) with just the prefactors differing by less than 25%. In addition, we obtained a relation for the oscillation amplitude $|\phi_1| \propto \sqrt{\kappa}$ at the bifurcation threshold.

F. Simulated Device Data for $\kappa_c \gg \kappa_s$

In Supplementary Figure 5 we present simulated device data for a case where the input-output coupling rate κ_c is approximately an order of magnitude larger than the dissipation rate κ_s. This is done so that the dissipation $\kappa_s/2\pi = 30$ MHz, and internal losses $\kappa_i/2\pi = 60$ MHz are kept the same as in the measured device, but where the input/output coupling is increased to $\kappa_c/2\pi = 240$ MHz. All the other parameter values are the same as for the measured device. Here the linewidth $\kappa/2\pi = 300$ MHz is an order of magnitude greater than the sensor dissipation κ_s. Despite being an order of magnitude away from the matching condition, the calculations still predict near-unity signal strength when operating the device at an input power just below the onset of the nonlinear

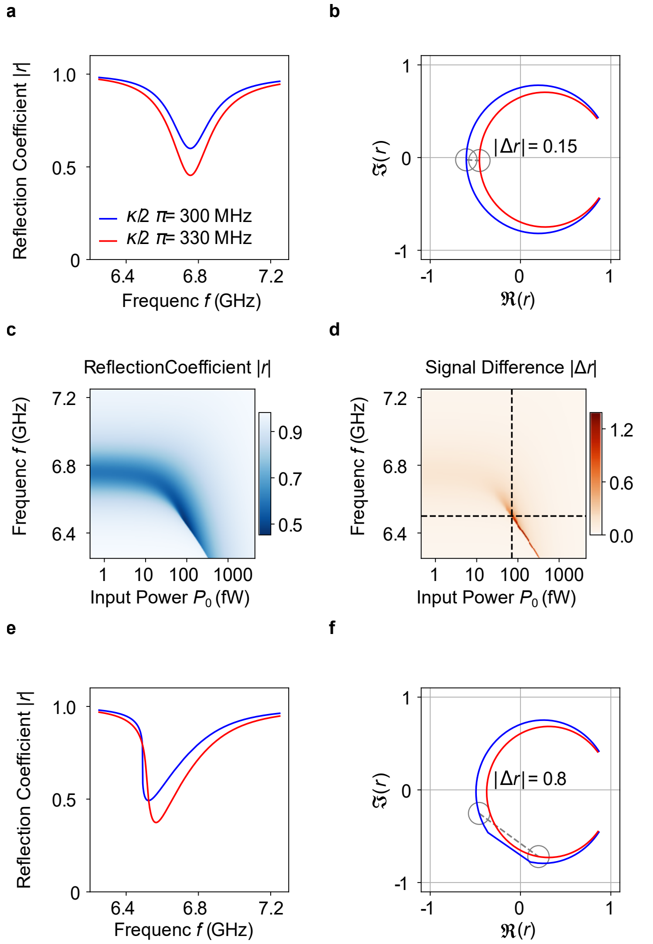

Supplementary Figure 5. Simulated response for larger input/output coupling Results from simulating a device with $\kappa_c/2\pi = 240$ MHz, $\kappa_i/2\pi = 60$ MHz and $\kappa_s/2\pi = 30$ MHz. **a** The low-power amplitude response as a function of the frequency f. **b** The corresponding plot of the complex response along with the maximum signal difference $|\Delta r|$. **c** & **d** The reflection coefficient $|r|$ & signal difference $|\Delta r|$ as a function of the frequency f and input power P_0. **e** The high-power amplitude response as a function of the frequency f, at the power $P_0 = 71$ fW, indicated by the vertical line in panel **d**. **f** The corresponding plot of the complex response along with the maximum signal difference $|\Delta r|$ at low power.

bifurcation. Here, the onset power where $\Delta\omega_K = -\kappa$ is $P_0 = 71$ fW, with a corresponding voltage amplitude $V = 60$ μV.

With this coupling configuration, and a doubling of the number of junctions in the resonator, one could quadruple the input power without consequence to the sensor contrast. Additionally, our measurement setup has ~ 3 dB of cable losses between the device and the first amplifier at 4 K, which could be replaced with superconducting cables to increase the signal strength at the amplifier input, granting another factor 2 in speed. With these considerations, achieving a measurement time of 10 ns is deemed realistic.

This demonstrates the prospects of a better optimized device to reach sub-10 ns response time, permitting charge detection well-within the dephasing times of charge- and spin qubits alike.

[1] H. Havir, S. Haldar, W. Khan, S. Lehmann, K. A. Dick, C. Thelander, P. Samuelsson, and V. F. Maisi, Quantum dot source-drain transport response at microwave frequencies, Phys. Rev. B **108**, 205417 (2023).

[2] M. Göppl, A. Fragner, M. Baur, R. Bianchetti, S. Filipp, J. M. Fink, P. J. Leek, G. Puebla, L. Steffen, and A. Wallraff, Coplanar waveguide resonators for circuit quantum electrodynamics, J. Appl. Phys. **104**, 113904 (2008).

[3] S. Andersson, H. Havir, A. Ranni, S. Haldar, and V. F. Maisi, High-impedance microwave resonators with two-photon nonlinear effects, Nat. Commun. **16**, 552 (2025).

[4] P. Bhupathi, P. Groszkowski, M. P. DeFeo, M. Ware, F. K. Wilhelm, and B. L. T. Plourde, Transient dynamics of a superconducting nonlinear oscillator, Phys. Rev. Appl. **5**, 024002 (2016).

[5] K. Johannessen, The duffing oscillator with damping for a softening potential, Int. J. Appl. Comput. Math. **3**, 3805 (2017).

[6] Q.-M. Chen, M. Fischer, Y. Nojiri, M. Renger, E. Xie, M. Partanen, S. Pogorzalek, K. G. Fedorov, A. Marx, F. Deppe, and R. Gross, Quantum behavior of the duffing oscillator at the dissipative phase transition, Nature Commun. **14**, 2896 (2023).

[7] G. M. Moatimid, T. S. Amer, and W. S. Amer, Dynamical analysis of a damped harmonic forced duffing oscillator with time delay, Sci. Rep. **13**, 6507 (2023).

[8] J. Koch, T. M. Yu, J. Gambetta, A. A. Houck, D. I. Schuster, J. Majer, A. Blais, M. H. Devoret, S. M. Girvin, and R. J. Schoelkopf, Charge-insensitive qubit design derived from the Cooper pair box, Phys. Rev. A **76**, 042319 (2007).

[9] G. Kirchmair, B. Vlastakis, Z. Leghtas, S. E. Nigg, H. Paik, E. Ginossar, M. Mirrahimi, L. Frunzio, S. M. Girvin, and R. J. Schoelkopf, Observation of quantum state collapse and revival due to the single-photon Kerr effect, Nature **495**, 205 (2013).

[10] I.-C. Hoi, A. F. Kockum, T. Palomaki, T. M. Stace, B. Fan, L. Tornberg, S. R. Sathyamoorthy, G. Johansson, P. Delsing, and C. M. Wilson, Giant cross–Kerr effect for propagating microwaves induced by an artificial atom, Phys. Rev. Lett. **111**, 053601 (2013).

[11] T. Yamaji, S. Kagami, A. Yamaguchi, T. Satoh, K. Koshino, H. Goto, Z. R. Lin, Y. Nakamura, and T. Yamamoto, Spectroscopic observation of the crossover from a classical duffing oscillator to a Kerr parametric oscillator, Phys. Rev. A **105**, 023519 (2022).

[12] S. Haldar, H. Havir, W. Khan, S. Lehmann, C. Thelander, K. A. Dick, and V. F. Maisi, Energetics of microwaves probed by double quantum dot absorption, Phys. Rev. Lett. **130**, 087003 (2023).

[13] F. R. Ong, M. Boissonneault, F. Mallet, A. Palacios-Laloy, A. Dewes, A. C. Doherty, A. Blais, P. Bertet, D. Vion, and D. Esteve, Circuit QED with a nonlinear resonator: AC-Stark shift and dephasing, Phys. Rev. Lett. **106**, 167002 (2011).
