# Supplementary material for: Near-unity charge readout signal in a nonlinear resonator without matching the sensor dissipation
Source: Nat Commun. 2026 Jul 2;17:5781. doi: 10.1038/s41467-026-75082-w (PMC13328590; doi:10.1038/s41467-026-75082-w)
Supplement: Supplementary file 2 — Transparent Peer Review file [file 41467_2026_75082_MOESM2_ESM.pdf]

nature portfolio

Peer Review File

Near-Unity Charge Readout Signal in a Nonlinear Resonator without Matching the Sensor Dissipation

Corresponding Author: Dr Harald Havir

This manuscript has been previously reviewed at another journal. This document only contains information relating to versions considered at Nature Communications.

This file contains all reviewer reports in order by version, followed by all author rebuttals in order by version.

Version 0:

Reviewer comments:

Reviewer #3

(Remarks to the Author)
I appreciate the authors' efforts in addressing the reviewers' comments and I am satisfied with the manuscript except for one point. I fully understand that an experimental demonstration of (ultra-)fast charge/conductance sensing in the time domain is constrained by the hardware configuration in the present study. However, the applicability of this technique to rapid charge detection is crucial for determining the relevance of the proposed matching approach. While the authors now discuss the expected response time in their rebuttal based on experimentally evaluated parameters in a rather conventional manner, I remain concerned about its validity. The response timescales of the bifurcation process are not incorporated at all in the considerations although the response time can be significantly delayed, for instance, near the threshold condition for a Duffing oscillator. I would like to ask the authors to strengthen or justify this part of the discussion and incorporate it in the manuscript.

Open Access This Peer Review File is licensed under a Creative Commons Attribution 4.0 International License, which

permits use, sharing, adaptation, distribution and reproduction in any medium or format, as long as you give appropriate credit to the original author(s) and the source, provide a link to the Creative Commons license, and indicate if changes were made.
In cases where reviewers are anonymous, credit should be given to 'Anonymous Referee' and the source.
The images or other third party material in this Peer Review File are included in the article’s Creative Commons license, unless indicated otherwise in a credit line to the material. If material is not included in the article’s Creative Commons license and your intended use is not permitted by statutory regulation or exceeds the permitted use, you will need to obtain permission directly from the copyright holder.
To view a copy of this license, visit https://creativecommons.org/licenses/by/4.0/

Reviewer #3 (Remarks to the Author):

Referee: I appreciate the authors' efforts in addressing the reviewers' comments and I am satisfied with the manuscript except for one point. I fully understand that an experimental demonstration of (ultra-)fast charge/conductance sensing in the time domain is constrained by the hardware configuration in the present study. However, the applicability of this technique to rapid charge detection is crucial for determining the relevance of the proposed matching approach. While the authors now discuss the expected response time in their rebuttal based on experimentally evaluated parameters in a rather conventional manner, I remain concerned about its validity. The response timescales of the bifurcation process are not incorporated at all in the considerations although the response time can be significantly delayed, for instance, near the threshold condition for a Duffing oscillator. I would like to ask the authors to strengthen or justify this part of the discussion and incorporate it in the manuscript.

Our response: Thank you for the comment. This is indeed a relevant question that we did not address in the previous revision round.

We bring up now this point, and the need for follow up experiments to test the obtainable speed increase in the main manuscript with the added text: *"In addition, the nonlinearities may decrease the response speed of the resonator, see Sect. D in the Supplemental materials for details. Further experiments are therefore needed for testing the achievable speed increase."*

As evident from the above addition, we also added a new section *"D. Estimation of the resonator response speed reduction from nonlinear effects"* to the supplemental materials. We estimate in this section now the speed reduction for the operation point of Fig. 3 of the main manuscript. The slowed response time of the resonator is reflected as a lower κ_c value in the second term of the equation of motion, Eq. (5) in the supplemental materials. This term describes the dissipation rate of the resonator. When one does not make approximations to this term, it will have an additional factor that reads

$$\left(1 - \frac{C_r}{C_c}\left(1 - \cos(\phi)\right) + \frac{C}{C_c}\left(1 - \omega^2/\omega_r^2\right)\right). \tag{1}$$

Here the first term is the leading order value used in the calculations and the other two terms are corrections to that. With the phase amplitude $\phi = 0.1\pi$ of the operating point for the 80 ns readout, along with the used frequency of $\omega/2\pi = 6.593$ GHz, the value of κ_c is reduced by 5 % due to this term. We estimate that the resonator response time slows down by a similar amount, and thus does not change the response speed significantly. This estimation is made in the new section in the supplemental information, and is backed up with four references that report similar transient response times in the nonlinear regime as in the linear one. We find it therefore likely that the response time is not significantly degraded. But as said in the main manuscript now: Future experiments are to be done to test this experimentally.

Finally, a table of device parameters has been added to the end of the supplemental section C containing the device parameters used in the numerical calculations to aid in reproducing the results of the numerical calculations.
